# Ex vivo cardiovascular magnetic resonance measurements of right and left ventricular mass compared with direct mass measurement in excised hearts after transplantation: a first human SSFP comparison

**DOI:** 10.1186/s12968-014-0074-0

**Published:** 2014-10-01

**Authors:** Nicholas J Farber, Sahadev T Reddy, Mark Doyle, Geetha Rayarao, Diane V Thompson, Peter Olson, Jerry Glass, Ronald B Williams, June A Yamrozik, Srinivas Murali, Robert WW Biederman

**Affiliations:** Gerald McGinnis Cardiovascular Institute, Allegheny General Hospital, Pittsburgh, PA USA; Department of Pathology, Allegheny General Hospital, Pittsburgh, PA USA

**Keywords:** Cardiovascular magnetic resonance, Cardiac mass, LV and RV mass, Explanted hearts, Steady-state free precession

## Abstract

**Background:**

CMR is considered the ‘gold standard’ for non-invasive LV and RV mass quantitation. This information is solely based on gradient-recalled echo (GRE) sequences while contrast dependent on intrinsic T1/T2 characteristics potentially offers superior image contrast between blood and myocardium. This study aims, for the first time in humans, to validate the SSFP approach using explanted hearts obtained from heart transplant recipients. Our objective is establish the correlation between and to validate steady-state free precession (SSFP) derived LV and RV mass vs. autopsy mass of hearts from cardiac transplants patients.

**Methods:**

Over three-years, 58 explanted cardiomyopathy hearts were obtained immediately upon orthotopic heart transplantation from the OR. They were quickly cleaned, prepared and suspended in a saline-filled container and scanned ex vivo via SSFP-SA slices to define LV/RV mass. Using an automatic thresholding program, segmentation was achieved in combination with manual trimming (ATMT) of extraneous tissue incorporating 3D cardiac modeling performed by independent and blinded readers. The explanted hearts were then dissected with the ventricles surgically separated at the interventricular septum. Weights of the total heart not excluding papillary and trabecular myocardium, LV and RV were measured via high-fidelity scale. Linear regression and Bland-Altman plots were used to analyze the data. The intra-class correlation coefficient was used to assess intra-observer reliability.

**Results:**

Of the total of 58 explanted hearts, 3 (6%) were excluded due to poor image quality leaving 55 patients (94%) for the final analysis. Significant positive correlations were found between total 3D CMR mass (450 ± 111 g) and total pathology mass (445 ± 116 g; r = 0.99, p < 0.001) as well as 3D CMR measured LV mass (301 ± 93 g) and the pathology measured LV mass (313 ± 96 g; r = 0.95, p < 0.001). Strong positive correlations were demonstrated between the 3D CMR measured RV mass (149 ± 46 g) and the pathology measured RV mass (128 ± 40 g; r = 0.76, p < 0.001). The mean bias between 3D-CMR and pathology measures for total mass, LV mass and RV mass were: 3.0 g, -16 g and 19 g, respectively.

**Conclusions:**

SSFP-CMR accurately determines total myocardial, LV and RV mass as compared to pathology weighed explanted hearts despite variable surgical removal of instrumentation (left and right ventricular assist devices, AICD and often apical core removals). Thus, this becomes the first-ever human CMR confirmation for SSFP now validating the distinction of ‘gold standard’.

## Background

An accurate and reproducible measurement of both left ventricular mass (LVM) and right ventricular mass (RVM) is important for cardiac risk stratification. Currently, cardiovascular magnetic resonance (CMR) imaging is considered the ‘gold standard’ for non-invasive measurement of left and right ventricular volume, mass and by extension LVEF and RVEF [1,2]. CMR offers a spatially defined 3D data set that spans the entire heart. The volume and mass measurements afforded by CMR are thus independent of cardiac geometric assumptions and are a major advantage over 1D and 2D echocardiography. This issue is particularly important for the RV, as its complex, crescentic geometry is challenging for mathematical modeling, placing increased importance on accurate mass calculations free of geometric assumptions [3]. A variety of prior studies have demonstrated CMR’s superior accuracy and reproducibility in assessing ventricular volumes over other imaging modalities, including M-mode and 2D echocardiography [4-9]. While the assessment of left ventricular mass by real-time 3D echocardiography has overcome some of the geometric assumptions required of 2D echocardiography methods, its clinical utility is still limited when characterizing the right ventricle and in patients with a poor acoustic window [10].

An ideal way to assess the mass calculation capabilities and intrinsic accuracy of any imaging modality is to independently compare the imaging-derived mass to a corresponding autopsy mass. In this way Devereux and Reichek in 1977 performed their seminal work comparing echocardiography with autopsy validation to derive an imaging-based left ventricular mass equation [11]. Their approach is closely replicated herein for the first time with CMR. Briefly, their intrepid work involved identifying terminally ill patients and obtaining research echocardiograms prior to autopsy, then comparing these *in vivo* ventricular mass measurements to the autopsy measurements. Before then, the determination of echocardiographic LV mass calculations was only postulated, not validated by autopsy. Currently, the recognition of CMR as the gold standard for non-invasive left and right ventricular mass calculation is largely based on animal and phantom data; the only human autopsy studies, as explained below, do not utilize the most recent pulse sequences, undermining the credibility of CMR as the *true* ‘gold standard’ [12-15]. Moreover, these older studies, in addition to small sample sizes, employed spin-echo or gradient recalled echo (GRE) pulse sequences for image acquisition. GRE sequences had numerous well defined deficiencies including flow-related enhancement and reliance on flow to define cardiac boundaries with consequent overestimation of mass and underestimation of volume. Despite these limitations, GRE served remarkably well for several decades. However, as is well known, the newer steady-state free precession (SSFP) approach not only has become the workhorse sequence for CMR due to its intrinsic T1/T2 contrast attributes but has become the *de facto* gold standard for measurement of LV mass due to its inherent resolution. Thus, there is an obligate need to reassess and validate CMR using SSFP sequences as the current gold standard for ventricular mass quantification.

It should be noted that when validated with animal data, SSFP *was* shown to be the scanning sequence that yielded CMR-derived ventricular mass closest to the autopsy mass [16]. Thus, the next logical step is to validate mass acquisition by CMR’s SSFP sequences in human hearts by autopsy. Therefore, the aim of our study was to establish a correlation between CMR derived left and right ventricular mass and autopsy-derived ventricular mass of *ex vivo* hearts from heart transplants using the SSFP sequence, thereby determining the credibility of CMR as the gold standard for non-invasive left and right ventricular mass calculation. However, today’s ability to replicate the autopsy approach used by Devereux and Reichek is more constrained than in the past as the more restrictive research environment, with increased protection of patients and institutional review boards, makes it all but impossible to duplicate their methods. Nonetheless, there are alternative ways, which are more sensitive to patients’ rights and would achieve similar results to what was done in the 1970’s, to execute this study. Our solution was to use the diseased hearts (explanted) from orthotopic heart transplantation as the source of hearts for imaging. Naturally, this approach removes the need and arguable issue of *in vivo* scanning of terminally ill patients just to establish a *bona fide* gold standard.

## Methods

**Figure 1:**
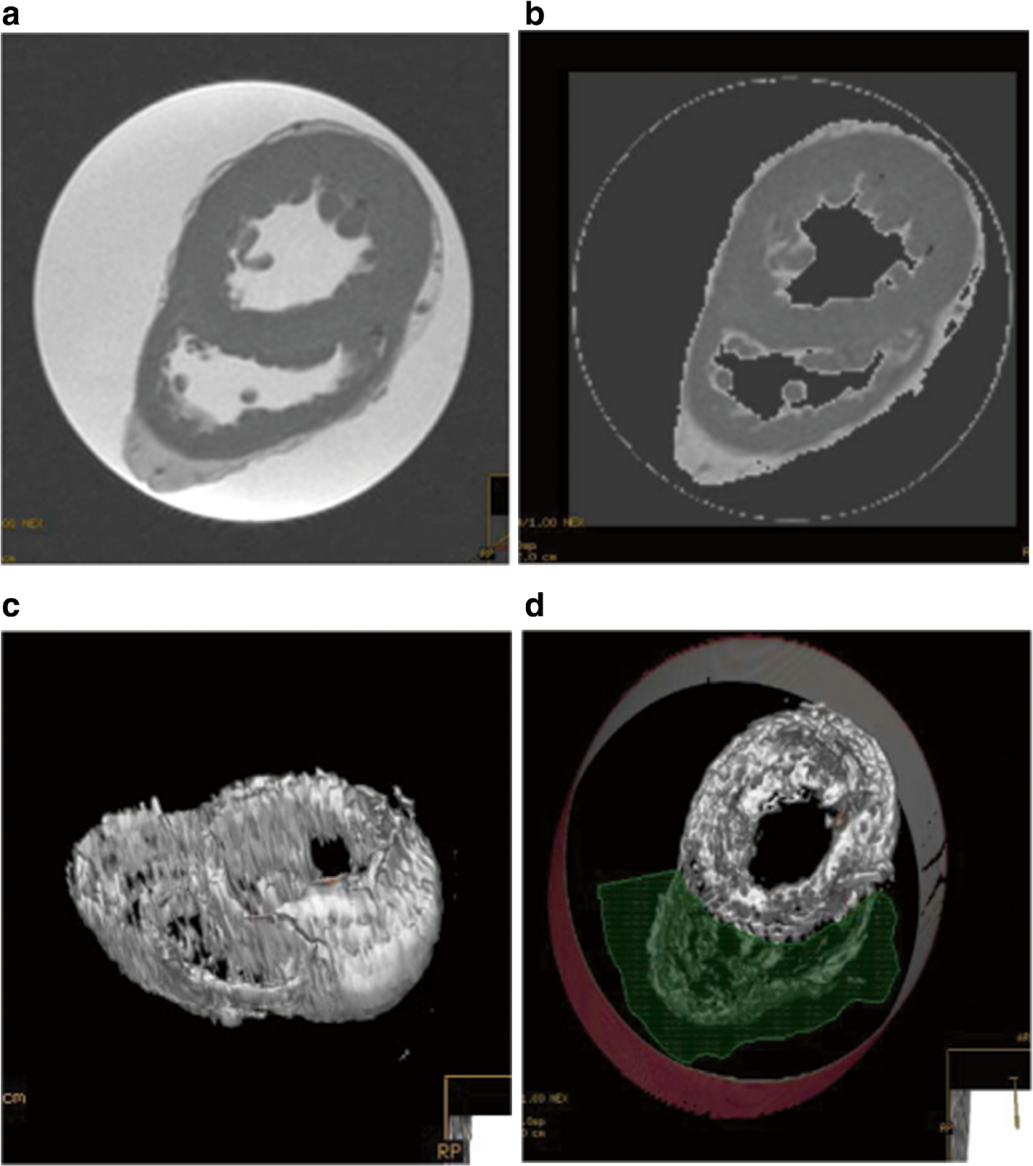
**Summary of ATMT steps. a)** Cross-sectional view of an explanted heart submerged in a cylindrical water bath. **c)** Application of upper and lower intensity thresholds to eliminate bulk of signal associated with the water bath. **c)** By successively removing extra-cardiac features, the 3D heart voxels are isolated. **d)** To isolate the left ventricle an irregular region encompassing the right ventricle is manually identified for removal.

In this prospective IRB approved study, explanted hearts from heart transplant recipients (n = 58) were obtained at the time of orthotopic transplantation within five minutes of harvest. The hearts were prepared by trimming atria and vascular components leaving only LV and RV myocardium including papillary and trabecular tissue. The hearts were submerged in a saline filled plastic container and CMR imaging was performed (GE, Milwaukee, WI; 1.5 T) using standard steady-state-free-precession (SSFP) approach (TR/TE/flip angle 4.2/2.1/45; FOV 350 mm, matrix 256 × 256, scan percentage 100%.) with slices oriented parallel to the LV short axis, applied contiguously from base to apex (slice thickness 8 mm, gap 0 mm). A birdcage design neurovascular coil was used to receive the signal. Using an image analysis program available with our scanner (Medis, The Netherlands), Automatic Thresholding with Manual Trimming (ATMT) was performed on the CMR set of images to isolate and contour cardiac tissue. Specifically, the ATMT approach was utilized given the complexity of the heart analysis with necessary removal of LVAD and/or RVAD apical cores, lateral wall surgical frozen section removal and AICD/PM lead extractions that markedly and exceptionally non-uniformly destroyed the primary myocardial architecture and geometry so critical for natural and heuristic manual endocardial and epicardial contour analysis. Formally, ATMT involved the automatic setting of an upper and lower myocardial signal threshold such that cardiac tissue was, via signal intensity, isolated from the surrounding saline bath signal from which the heart was suspended for imaging. Secondly, the signal was rendered as a 3D structure, which could be rotated within the viewing station permitting more user-controlled defined ROI contours to the often no-longer intuitive LV/RV geometry. Using a succession of cuts applied to the 3D model, non-cardiac tissue signal (saline which had near identical signal intensity to fat, distortion artifacts as apex approached the container and retained air) was systematically removed leaving a 3D model for the independent and blinded reader facilitating final manual contouring. In short: assisted manual contouring to permit formal comparison of CMR mass to autopsy mass whereas the former is and the latter is not dependent on uniformity of structure (Figure 1).

Initially, the whole cardiac structure was isolated, and using the features of the processing software, the volume of myocardium was determined. Next, this was converted into grams by multiplication of the density of myocardium, taken as 1.055 g/mm^3^. After recording the total cardiac volume, the same model was further operated on to remove the RV structure by use of a series of digital ‘cuts’ applied to the 3D model. The remaining tissue (typically trimmed to a semi elliptical structure) was measured and taken as the LV or RV volume. Following performance of the CMR examination, the explanted hearts were returned to the Pathology Lab, where the whole heart was weighed using a high-fidelity scale. The pathologist then formally separated the RV tissue using scissors, and the RV and LV tissue were weighed separately. These steps understandably required intricate coordination between the transplant surgeons, pathologists and the CMR Laboratory day or night given the natural nuances of obtaining donor hearts. Finally, ten random explant hearts were also reanalyzed to establish the intra-observer variability by the same observer (GR) using the exact same technique. The reanalysis was blinded and took place 4 months after the initial analysis to avoid memory bias.

### Statistical analysis

Continuous variables were expressed as mean + standard deviation and compared using the independent samples t test. Categorical variables were expressed as counts and percentages. Linear regressions were performed to assess the correlation of cardiac mass weights between the SSFP-CMR method and ex-vivo heart autopsy method. Bland-Altman plots were used to determine the bias and 95% limits of agreement between methods. A two-tailed p-value <0.05 was considered to indicate statistical significance. The intra-class correlation coefficient (ICC) was calculated to assess intra-observer reliability between measures of 3D MRI volumes made by a single rater using the ATMT method. The ICC model used was two-way mixed and the type was absolute agreement. The values of ICC range between 0 and 1.0, with values closer to 1.0 representing stronger reliability. Portney and Watkins [17] suggested that ICC value above 0.75 indicate good reliability, those between 0.5 and 0.75 indicate moderate reliability, and those below .50 indicate poor reliability). Data was analyzed using IBM SPSS Statistics, version 20.0 (IBM Corporation, Armonk, NY).

## Results

A total of 58 explanted hearts were imaged; 3 (5%) were excluded due to poor image quality (peri-surgical destruction) leaving 55 (95%) for the final analysis. Of the 55 explanted hearts, 39 (71%) were men, and 16 (29%) were women (mean age 55 ± 11 years, range 23 to 73 years). A significant proportion of the explanted hearts belonged to ischemic (47%) and non-ischemic heart disease (36%) patients (Table 1). Instrumentation was present in all but two patients comprised of AICD and or PM (93%), LVAD (60%), RVAD (15%) and/or RVAD/LVAD (12%). All (100%) patients had LV and RV (42%) myocardial surgical resection of varying amounts averaging 3x3cm. The average time from Transplant Suite to Pathology Department was 25 ± 5 and from Pathology to CMR notification was 30 ± 5 minutes. CMR preparation time (RB or RW) was 10 ± 2 minutes minimizing desiccation and impending formaldehyde preservation. The processing time per image series was approximately 8–10 min; not significantly different than standard manual contouring. The total time the hearts were rerouted to the CMR facility was < 60 minutes.

**Table 1:** Heart transplant demographics

**Demographics**	**Number (55)**
Age (years)	55 ± 11
Female	16 (29%)
Male	39 (71%)
Ischemic Cardiomyopathy	26 (47%)
Hypertrophic Cardiomyopathy	2 (4%)
Miscellaneous Etiologies	3 (6%)
Non-ischemic Cardiomyopathy	20 (36%)
Re-transplantation	4 (7%)

**Figure 2:**
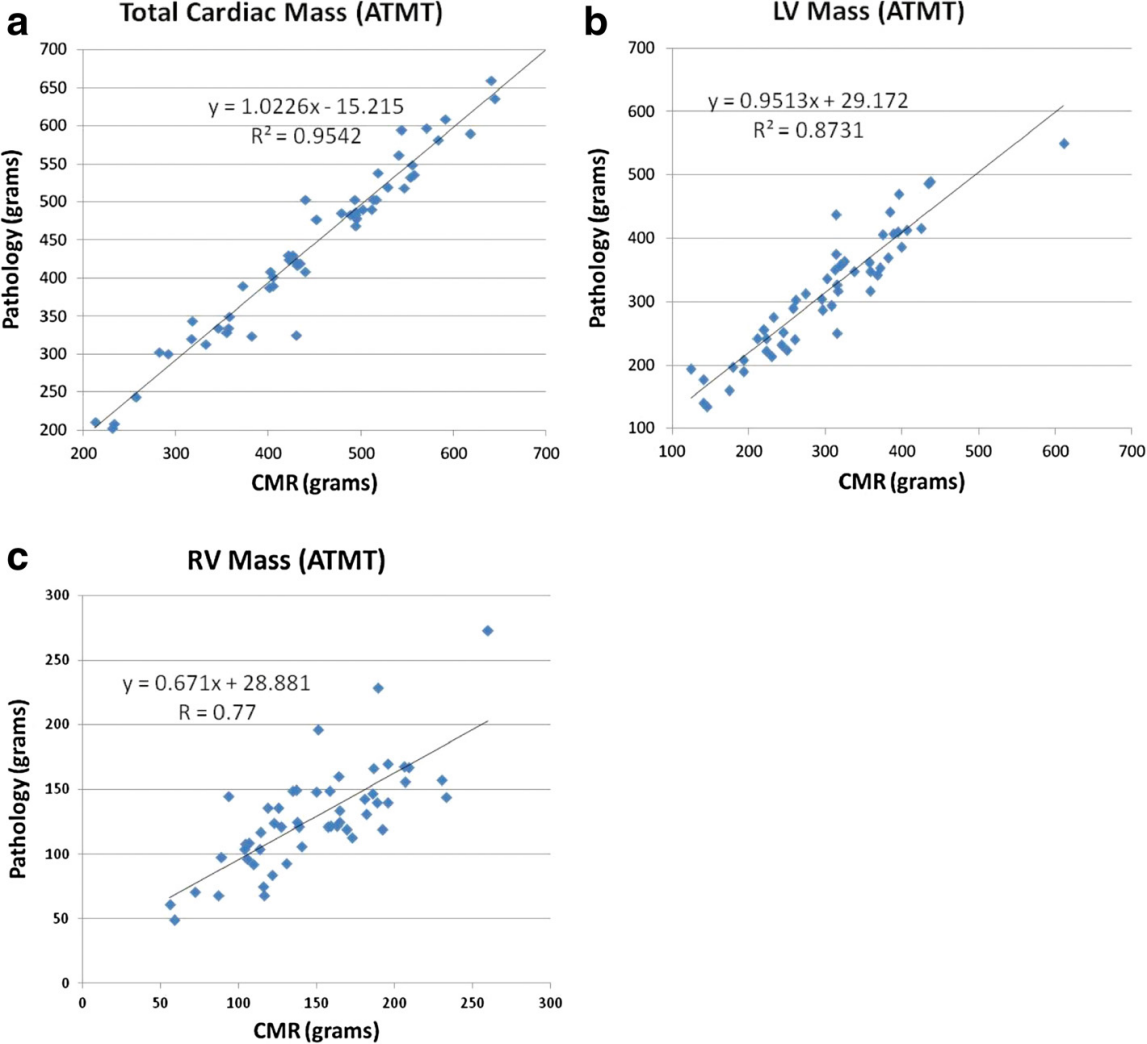
**Linear Regression Analysis CMR vs. Pathology Mass. a**: comparison of combined ventricular, left and right ventricular mass measurement by cardiac magnetic resonance imaging with autopsy/pathology mass. **b**: Linear regression analysis: comparison of left ventricular (LV) mass measurement by cardiac magnetic resonance imaging with autopsy/pathology mass. **c**: Linear regression analysis: comparison of right ventricular (RV) mass measured by cardiac magnetic resonance imaging with autopsy/pathology mass.

**Figure 3:**
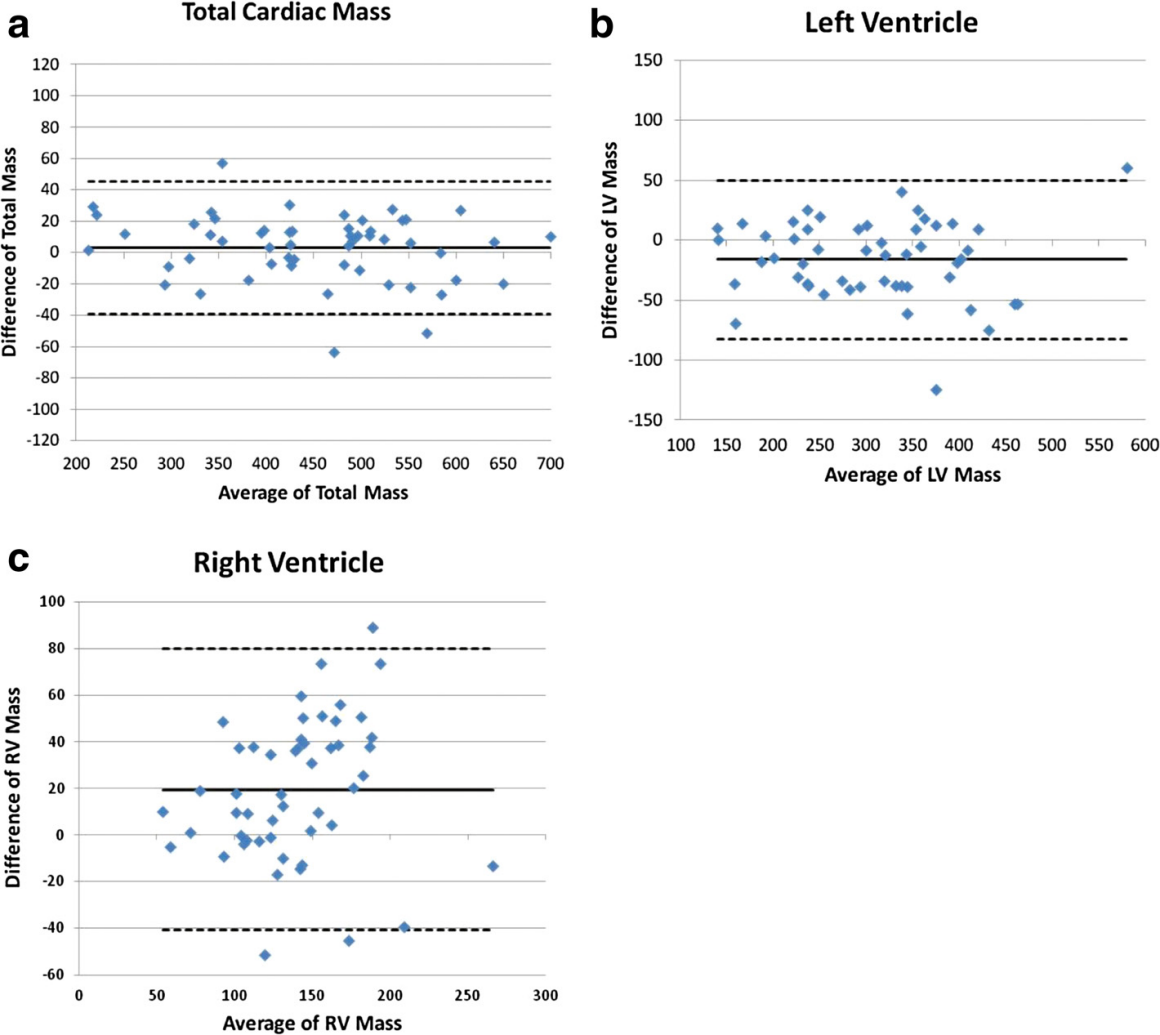
**Bland-Altaman plots.** **a)** Combined ventricular mass by CMR vs. pathology mass. **b)** LV mass by MRI vs. pathology mass, and **c)** RV mass by MRI vs. pathology mass.

Extremely strong positive correlations between total CMR 3D mass (450 ± 111 g) and total pathology mass (445 ± 116 g; r = 0.99, p < 0.001) and the 3D CMR measured LV mass (301 ± 93 g) versus the pathology measured LV mass (313 ± 96 g; r =0.95, p < 0.001). Strong positive correlation was demonstrated between the 3D CMR measured RV mass (149 ± 46 g) and the pathology measured RV mass (128 ± 40 g; r = 0.77, p < 0.001) R = 0.77 (Table 2). The equations y = 1.023 × – 15.2b (r = 0.99); y = 0.9513 × + 29.172 (r = 0.95); y = 0.671 × + 28.881 (r = 0.77) regressed the total mass, LV mass and RV mass respectively (Figure 2). The average bias between 3D CMR and pathology measures for total mass, LV mass and RV mass were: 3.0 g (95% limits of agreement (LOA) -39 to 46), -16 (-82 to 50), and 19 g (-41 to 80), respectively. The differences between CMR measured mass and pathology mass for all three indices were illustrated in Bland-Altman plots (Figure 3).

**Table 2:** Cardiac mass weight cmr vs. pathology

**Parameter**	**Total cardiac mass (g)**	**Left ventricular mass (g)**	**Right ventricular mass (g)**
**CMR mass (g)**	450 ± 111	301 ± 93	149 ± 46
**Pathology mass (g)**	445 ± 116	313 ± 96	128 ± 40
**R Value**	0.99	0.95	0.77

The intraclass correlation coefficient (ICC) was calculated for ten randomly selected hearts (15%) to assess intra-observer reliability between measures of 3D MRI volumes made by a single rater using the ATMT method. For 3D MRI total volume measured in October 2013 versus the February 2014 measurement was 0.990 (95% confidence interval, 0.958 to 0.998). For 3D MRI LV volume the ICC measurement was 0.986 (95% confidence interval, 0.948–0.997). For 3D MRI RV volume the ICC measurement was 0.730 (95% confidence interval, 0.234–0.925).

## Discussion

The ideal method for quantification of ventricular mass should be fairly quick, non-invasive, highly accurate and reproducible and applicable to each ventricle. In our study, we were able demonstrate that total ventricular mass, LV and RV mass obtained by CMR showed excellent correlation with histopathology mass obtained on the explanted hearts (r = 0.99, 0.95, and 0.77, respectively). This study demonstrates that accurate and reproducible assessment of both left and right ventricular masses can be efficiently obtained non-invasively using CMR’s SSFP sequences, as compared to weighed, explanted human donor hearts. Most importantly, this is the final validation step so critical in unequivocally depicting CMR as the true ‘gold standard’ that heretofore had been remarkably accepted *de facto* given the distinct and glaring absence of such a validation step.

**Figure 4:**
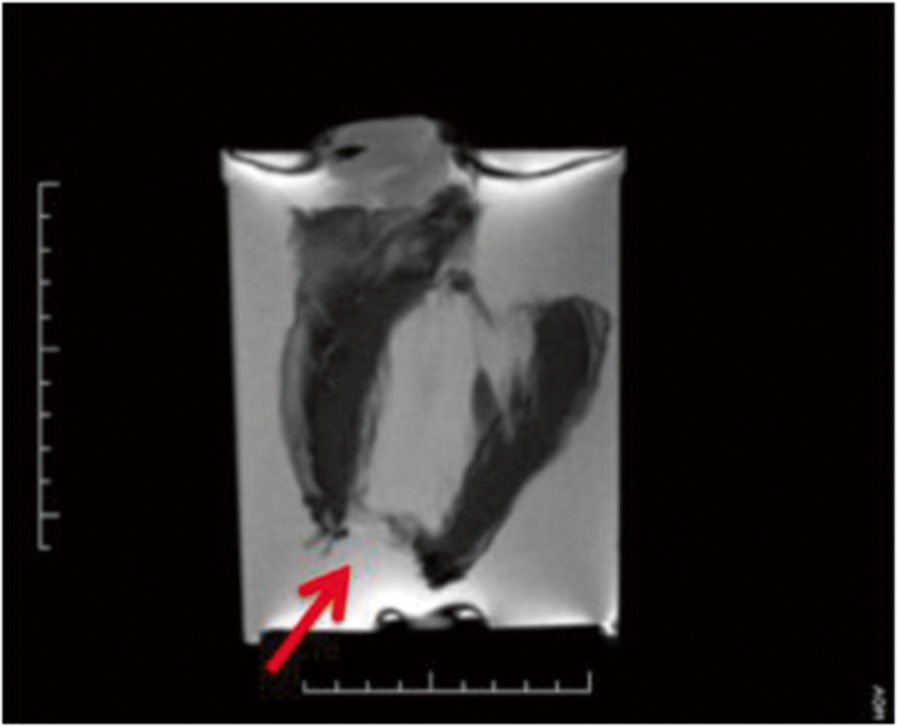
**Long axis view of suspended explanted heart featuring destruction of the anterior wall and missing apex due to previous LVAD instrumentation.**

Considered not possible in the current era, a confirmation similar to the manner in which Devereux and Reichek first validated echocardiographic mass (Penn Equation) required a resourceful contemporary approach. Awaiting a person’s imminent death is considered taboo by stateside IRB’s and justifiably so. Arriving on a natural ready supply of explanted *ex vivo* hearts, while not *in vivo*, was the crucial element and an aspect that fundamentally required an exceptional synchronization amongst the Cardiac Transplant Service, Pathology and CMR Laboratory at any time. These invaluable results were obtained despite several possibly obfuscating variables. Chiefly, the cardiomyopathic, ex-planted hearts all possessed variable mechanical instrumentation before transplant, including left and right ventricular assist devices (LVAD/RVAD) and implantable cardioverter-defibrillators (AICD) and/or pacemakers. The removal of this instrumentation upon explanation often led to partial or full apical core removal, leaving an apical interface that was far more difficult to assess when drawing epicardial and endocardial contours (Figure 4). As well, a separate lateral wall surgical section was always removed for Surgical Pathology for confirmation of etiology of the cardiomyopathy. Thus, there was a substantial inherent destruction and radical distortion of the primary variable, potentially leading to wholesale inability to achieve reliable correlation. The fact that the whole heart, left and right ventricular masses still correlated extremely well with the weighed masses is a further testament to the accuracy of CMR; an even higher correlation is predicted when the natural boundaries of the heart, with the apex fully intact, provide an enhanced guide for contouring as in routine CMR imaging. A final factor to consider is the use of a cardiomyopathic patient population in this study. Ideally, a separate subgroup of non-cardiomyopathic hearts to establish a second correlation between CMR-derived and weighed ventricular LV and RV mass would be ideal. However, the use of less diseased, non- cardiomyopathic hearts would prove less problematic for epicardial and endocardial contouring, which would likely improve the accuracy of the CMR mass quantitation.

Establishing the accuracy and reproducibility of CMR measured cardiac mass has important implications for clinical practice and research. Prognostic significance of left ventricular mass regression was demonstrated by showing fewer rates of clinical end points in patients with lower LV mass during antihypertensive treatment [18]. An increase in left ventricular mass predicts a higher incidence of cardiovascular events including death and this information is in addition to that provided by the evaluation of traditional cardiovascular risk factors [19]. Thus, accurate measurement of LV mass by CMR has important clinical implications in terms of risk assessment and regression of LV mass in response to treatment. Interestingly, there are no studies in the literature that directly compared RV mass with prognosis. Our study further adds to the literature in demonstrating strong correlation between CMR measured RV mass and pathology mass. We believe this study provides a steppingstone for the use of RV mass as a prognostic stool as well as for assessing response to treatment in patients with pulmonary hypertension.

In a landmark paper, Bottini et al. demonstrated that detecting a 10 gram regression in LV mass with a power of 0.8 and p < 0.05 would require 550 patients using echocardiography but only require 17 patients using CMR. Thus, much smaller sample sizes can be used to detect the same change in LV mass, or smaller degrees of changes can be identified using the same sample size. Moreover, the precise measurement of ventricular mass has been proposed to more easily be utilized in a therapeutic approach, such as in LV mass regression with antihypertensive therapy [20]. The implications are important in that therapeutic efficacy, risk stratification and general accuracy can only be obtained when an exacting method is available. In this study, linear regression analysis indicated excellent correlations as shown in Figure 2 for all three indices including total cardiac mass, LV mass and RV mass determined by using 3D CMR with ATMT technique and true LV mass obtained at autopsy on explanted hearts. The differences between CMR measured mass and pathology mass including systemic and random differences for all three indices were illustrated in the Bland-Altman plots (Figure 3). The bias between CMR measured mass and pathology mass were low and not systematic.

An important and controversial consequence of this work has emerged. Namely does the conventional long accepted dogma for cardiac mass quantification *excluding* certain mass (papillary and trabecular mass) need to be discarded? It is well known that papillary muscles have the same thickness as the left ventricular free wall. Additionally, in patients with LV hypertrophy there is a proportional hypertrophy of the papillary muscles [21]. In a study by Francois, *et al* [14] it was demonstrated that the most accurate estimates of the LV mass in animal models were obtained when the papillary muscles were included in the myocardium. Furthermore, when the papillary muscles were excluded from the LV mass, the values were significantly lower than pathology mass measured at autopsy. However, historically, it is interesting to note that in the initial validation of GRE the correlations were *improved* by excluding the papillary muscles and fine trabecular tissue, no doubt due to the well-known flow-related enhancement that occurred with GRE causing overestimation, not underestimation. This then triggered the venerable mathematical regression correction we now use today. Interestingly, despite this knowledge, the modern and long-lauded SSFP sequences, devoid of this artifact, has not sparked a plea for reconsideration of the general approach of excluding papillary and trabecular tissue. Similar to the previous SSFP animal studies, our study *incorporated* papillary muscles and trabecular mass by using the ATMT method in calculating total LV mass while obtaining near unity with true autopsy mass. Further, the new regression equation now includes, not excludes, papillary and trabecular mass suggesting that our Society should now incorporate both into our manual contouring approaches. In conclusion, to our knowledge this study represents the first human modern CMR versus autopsy validation of human ventricular mass, and fairly emulates the intrepid days of the initial validation of the Penn Formula by Devereux and Reichek in 1977.

### Study limitations

Our chief limitation is that CMR scans were performed on *ex-vivo* hearts that were suspended in normal saline and these specimens obligatorily do not suffer from temporal resolution issues encountered in *in-vivo*, live, beating hearts. The contribution of this temporal blur is felt to be far less than the inexactitude of the underestimation encountered by systematically excluding important myocardium. It should also be noted that the initial premise of our study was to image patients referred for heart transplant via CMR, but as our center is a large tertiary/quaternary referral base, only the rare patient presented without an AICD. None of the hearts in our study had normal pathology and, in addition, significant numbers of explanted hearts (98%) possessed variable mechanical instrumentation before transplantation (e.g. LVADs, RVADs, and AICDs) which confounded natural contouring. The removal of this instrumentation upon explanation often led to partial or full apical (often biventricular) core removal, leaving an apical interface that was more difficult to assess when drawing epicardial and endocardial contours. Yet, despite the high prevalence of patients with prior mechanical instrumentation and the attendant difficulties of imaging these hearts, we still found excellent correlations between CMR and autopsy myocardial mass which supports the value of our approach. Further, that the RV correlations were less than the LV correlations might be expected due to the disproportionate effect of loss of RV contours with surgical manipulation. Less expected however was the effect in which the now deflated heart post-explant lost its RV/septal margins making separation of RV and LV less precise. In that the whole heart demonstrates near identity with autopsy weight *prior* to separation attests to the fidelity of the approach mitigating the absolute process itself. Finally, explanted hearts in this study were imaged at higher resolution than *in-vivo* hearts. For the purpose of the study, a limited separate analysis showed no significant difference when measured at conventional resolution.

## Conclusions

SSFP-CMR accurately determines total myocardial, LV and RV masses as compared to weighed explanted hearts, despite variable surgical removal of instrumentation (LVAD/RVAD, AICD’s and often apical core removals) in a transplant population. Thus, while GRE was the *original* ‘gold standard’ for LV mass, SSFP, despite its current universal acceptance as the ‘*de facto*’ gold standard’, is now formally validated in this autopsy study. Further, the regression equation now includes, not excludes, papillary and trabecular mass supporting the notion that our Society should consider incorporating both into standard cardiac measurements.

## Abbreviations

AICD: Automatic implantable cardioverter defibrillator
ATMT: Automatic Thresholding with Manual Trimming
CMR: Cardiovascular magnetic resonance imaging
GRE: Gradient-recalled echo
ICC: Intra-class correlation coefficient
LV: Left ventricle
LVAD: Left ventricular assist device
LVH: Left ventricular hypertrophy
LVM: Left ventricular mass
PM: Pacemaker
RV: Right ventricle
RVAD: Right ventricular assist device
RVM: Right ventricular mass
SSFP: Steady-state free precession
